# Further investigation of the role of HLA-DPB1 in adult Hodgkin's disease (HD) suggests an influence on susceptibility to different HD subtypes

**DOI:** 10.1038/sj.bjc.6690536

**Published:** 1999-07

**Authors:** G M Taylor, D A Gokhale, D Crowther, P J Woll, M Harris, D Ryder, M Ayres, J A Radford

**Affiliations:** Immunogenetics Laboratory, Department of Medical Genetics, St Mary's Hospital, Hathersage Road, Manchester, M13 0JH, UK; Departments of Medical Oncology, Pathology and Medical Statistics, Christie Hospital, Manchester, UK

**Keywords:** Hodgkin's disease, *HLA-DPB1*, HVR, susceptibility, resistance, polymorphic amino acid

## Abstract

It has been suggested in a number of studies that susceptibility to adult Hodgkin's disease (HD) is influenced by the *HLA class II* region, and specifically by alleles at the *HLA-DPB1* locus. Since HD is diagnostically complex, it is not clear whether different *HLA-DPB1* alleles confer susceptibility to different HD subtypes. To clarify this we have extended a previous study to type *DPB1* alleles in 147 adult HD patients from a single centre. We have analysed patients with nodular sclerosing (NS), mixed cellularity (MC) or lymphocyte predominant (LP) HD, and gender in relation to *HLA-DPBI* type, in comparison with 183 adult controls. The results confirmed previously reported associations of DPB1*0301 with HD susceptibility (relative risk (RR) = 1.42; 95% confidence interval (CI) 0.86–2.36) and DPB1*0201 with resistance to HD (RR = 0.49; CI 0.27–0.90). However, analysis by HD subtype and gender showed that *0301-associated susceptibility was confined to females with HD (RR = 2.46; CI 1.02–5.92), and *0201-associated resistance to females with NS-HD (RR = 0.28; CI 0.10–0.79). Susceptibility to NS-HD was also associated in females with *1001 (RR = 11.73; CI 1.32–104.36), and resistance with *1101 (RR = 0.08; CI 0.01–0.65). In contrast, susceptibility to LP-HD was associated in males with *2001 (RR = 32.14; CI 3.17–326.17), and to MC-HD with *3401 (RR = 16.78; CI 2.84–99.17). Comparison of *DPB1*-encoded polymorphic amino-acid frequencies in patients and controls showed that susceptibility to MC-HD was associated with Leucine at position 35 of *DPB1* (RR = 8.85; CI 3.04–25.77), Alanine-55 (RR = 15.17; CI 2.00–115.20) and Valine-84 (RR = 15.94; CI 3.55–71.49). In contrast, Glutamic acid 69 was significantly associated with resistance to MC-HD (RR = 0.14; CI 0.03–0.60). Certain *DPB1* alleles and individual DPβ1 polymorphic amino acid residues may thus affect susceptibility and resistance to specific HD subtypes. This may be through their influence on the binding of peptides derived from an HD-associated infectious agent, and the consequent effect on immune responses to the agent. © 1999 Cancer Research Campaign


*British Journal of Cancer* (1999) **80**(9), 1405–1411
© 1999 Cancer Research Campaign
Article no. bjoc.1999.0536

### Further investigation of the role of HLA-DPB1 in adult Hodgkin's disease (HD) suggests an influence on susceptibility to different HD subtypes

**GM Taylor[1], DA Gokhale[1], D Crowther[2], PJ Woll[2,*], M Harris[3], D Ryder[4], M Ayres[1] and JA Radford[2]**

[1]Immunogenetics Laboratory, Department of Medical Genetics, St Mary's Hospital, Hathersage Road, Manchester M13 0JH, UK; Departments of [2]Medical Oncology, [3]Pathology and [4]Medical Statistics, Christie Hospital, Manchester, UK

**Summary** It has been suggested in a number of studies that susceptibility to adult Hodgkin's disease (HD) is influenced by the *HLA class II* region, and specifically by alleles at the *HLA-DPB1* locus. Since HD is diagnostically complex, it is not clear whether different *HLA-DPB1* alleles confer susceptibility to different HD subtypes. To clarify this we have extended a previous study to type *DPB1* alleles in 147 adult HD patients from a single centre. We have analysed patients with nodular sclerosing (NS), mixed cellularity (MC) or lymphocyte predominant (LP) HD, and gender in relation to *HLA-DPBI* type, in comparison with 183 adult controls. The results confirmed previously reported associations of *DPB1*0301* with HD susceptibility (relative risk (RR) = 1.42; 95% confidence interval (CI) 0.86–2.36) and *DPB1*0201* with resistance to HD (RR = 0.49; CI 0.27–0.90). However, analysis by HD subtype and gender showed that **0301*-associated susceptibility was confined to females with HD (RR = 2.46; CI 1.02–5.92), and **0201*-associated resistance to females with NS-HD (RR = 0.28; CI 0.10–0.79). Susceptibility to NS-HD was also associated in females with **1001* (RR = 11.73; CI 1.32–104.36), and resistance with **1101* (RR = 0.08; CI 0.01–0.65). In contrast, susceptibility to LP-HD was associated in males with **2001* (RR = 32.14; CI 3.17–326.17), and to MC-HD with **3401* (RR = 16.78; CI 2.84–99.17). Comparison of *DPB1*-encoded polymorphic amino-acid frequencies in patients and controls showed that susceptibility to MC-HD was associated with Leucine at position 35 of *DPB1* (RR = 8.85; CI 3.04–25.77), Alanine-55 (RR = 15.17; CI 2.00–115.20) and Valine-84 (RR = 15.94; CI 3.55–71.49). In contrast, Glutamic acid 69 was significantly associated with resistance to MC-HD (RR = 0.14; CI 0.03–0.60). Certain *DPB1* alleles and individual DPβ1 polymorphic amino acid residues may thus affect susceptibility and resistance to specific HD subtypes. This may be through their influence on the binding of peptides derived from an HD-associated infectious agent, and the consequent effect on immune responses to the agent.

**Keywords:** Hodgkin's disease; *HLA-DPB1*; HVR; susceptibility; resistance; polymorphic amino acid

There have been numerous suggestions that Hodgkins disease (HD) may have an infectious aetiology (Vianna et al, 1971; Vianna, 1974; Alexander et al, 1991*a*; Mauch et al, 1993), though the epidemiological and diagnostic complexity of HD has hindered a complete understanding. Studies suggesting that the peak of HD cases in early adulthood HD resembles that of paralytic poliomyelitis in the pre-vaccine era (Gutensohn and Cole, 1977) support an aetiological pathway in which HD arises as a rare outcome of a common infection (Gutensohn and Cole, 1981). There is now extensive evidence to suggest that the infectious agent, at least in some cases, is Epstein–Barr virus (EBV) (Weiss et al, 1989; Wu et al, 1990; Khan et al, 1992; Jarrett, 1993; Oudejans et al, 1997). However, the ubiquitous distribution of EBV in the normal population, without accompanying disease (Miller, 1990), suggests that other factors, possibly of host origin, contribute to the aetiology of HD.

Immune responses to infectious agent causally associated with HD may be influenced by inter-individual genetic variations. Cell-surface heterodimers encoded by the *HLA class* II (*DR*, *DQ* and *DP*) loci play a pivotal role in the control of immune responses through the presentation of peptide antigens to T-cells (Brown et al, 1988; Germain, 1994; Hammer, 1995). A number of recent studies have suggested that alleles at the *HLA-DPB1* locus, the most centromeric of the three major HLA class II loci (*DR*, *DQ* and *DP*), may increase susceptibility and resistance to HD. Thus, *HLA-DPB1*0301* was found to be associated with susceptibility, and **0201* with resistance to HD (Bodmer et al, 1989; Tonks et al, 1992; Cesbron et al, 1993; Pellegris et al, 1993; Klitz et al, 1994; Oza et al, 1994).

The diagnostic complexity of HD and the excess of males with certain HD subtypes (Alexander et al, 1991*b*; Glaser and Jarrett, 1996) suggests that these two *DPB1* alleles (**0301*, **0201*) may not be the only ones to contribute to HD susceptibility and resistance. This view is supported by the fact that since DPβ1 subunits are made up of 2–3 polymorphic amino acid residues at each of six hypervariable regions (HVRs) in exon 2 (Bugawan et al, 1990), some alleles may have identical residues at certain HVRs, but not at others. In a previous study we alluded to a relationship between *DPB1* alleles and subtypes of HD (Taylor et al, 1996), but there were insufficient patients for a detailed analysis. To clarify this we have now extended the study to include 147 HD patients classified by subtype, gender and polymorphic amino acid alleles, and compared them with 183 controls.

*Received 14 April 1998*
*Revised 12 January 1999*
*Accepted 27 January 1999*

*Correspondence to:* GM Taylor

*Present address: Department of Medical Oncology, City Hospital, Nottingham, UK.

### MATERIALS AND METHODS

### Patients and controls

The patients consisted of 147 unrelated adult UK Caucasians with HD, aged between 15 and 75 years, from the North West of England. The series included 118 patients described in a preliminary report (Taylor et al, 1996) together with a further 29 patients. The patients all attended a single treatment centre (The Medical Oncology Clinic, Christie Hospital, Manchester, UK). Further details of the patients are given in Table 1. The control group consisted of 183 anonymous healthy adult blood donors, collected by the National Blood Transfusion Service (NBTS) in Manchester, UK. This compares with 92 adult controls in our previous study (Taylor et al, 1996). They were a randomly collected series consisting of 27.9% males, and 61.2% females. In 10% of cases, the sex of the donor was not specified by the NBTS for reasons of confidentiality. Gender analysis was thus confined to the controls where this information was available. The youngest control subject was 19 years and the oldest was 67 years, the mean and median ages being 41.4 and 42 years respectively.

### Histopathology

Review of tissue sections from diagnostic biopsies for all 147 patients was carried out in the Department of Histopathology at the Christie Hospital under the supervision of a cancer pathologist (MH). Assignment of HD subtype was performed in accordance with the Rye modification of the Lukes classification system (Lukes et al, 1966*a*, 1966*b*). For the purposes of this study, the HD cases were classified into nodular sclerosing (NS), mixed cellularity (MC), lymphocyte-predominant (LP), lymphocyte-depleted (LD) and unclassified. LP is included as a subtype of HD in this study for completeness and comparative purposes, even though LP is generally accepted to be a separate disease entity (Pan et al, 1996).

### *DPB1* genomic DNA amplification

Genomic DNA was extracted from the whole blood of patients and controls using established methods, and a 288-bp fragment of HLA-DPB1 exon 2 was amplified using the polymerase chain reaction (PCR) primers DPB1-5′ (GAG AGT GGC GCC TCC GC TCA T) and DPB1-3′ (GCC GGC CCA AAG CCC TCA CTC) using PCR conditions described previously (Taylor et al, 1996).

### *DPB1* typing with SSO probes

*DPB1* typing was carried by the method of Bugawan et al (1990) as described previously (Taylor et al, 1996) using 24 sequence-specific oligonucleotide (SSO) probes. DNA samples that could not be assigned a *DPB1* type following SSO hybridization were further analysed by direct DNA sequencing of PCR products.

### Allele assignment

*DPB1* alleles were assigned from patterns of SSO probe reactivities according to the XIth HLA workshop (Tait et al, 1992) and as detailed in the Report of the HLA Nomenclature Committee (Marsh, 1996). Polymorphic amino acids encoded by six HVRs situated in exon 2 were deduced from allele assignments as previously described (Taylor et al, 1996).

**Table 1** Diagnostic characteristics and gender of the 147 patients with Hodgkins disease included in the study

| | Males | | Females | | Total | |
|---|---|---|---|---|---|---|
| **Diagnostic subtype** | ***n*[a]** | **%[b]** | ***n*** | **%** | ***n*** | **%** |
| Nodular sclerosing (NS) | 47 | 50.5 | 43 | 79.6 | 90 | 61.2 |
| Lymphocyte predominant (LP) | 19 | 20.4 | 3 | 5.5 | 22 | 14.9 |
| Mixed cellularity (MC) | 18 | 19.3 | 6 | 11.1 | 24 | 16.3 |
| Lymphocyte depleted (LD) | 0 | 0 | 1 | 1.8 | 1 | 0.6 |
| Unclassified | 9 | 9.6 | 1 | 1.8 | 10 | 6.8 |
| Total | 93 | 100 | 54 | 100 | 147 | 100 |

[a]Number (n) of patients within each diagnostic subgroup. [b]Percentage (%) of patients in each subgroup.

**Table 2** Comparison of *HLA-DPB1*-typed HD patients ($n$ = 114) with a larger series of patients ($n$ = 897) from the same specialist centre

| **Patients** | **Present study** | **Total group** |
|---|---|---|
| Number of patients ($n$) | 114[a] | 897[b] |
| Median age (years) | 28.9 | 30.9 |
| Male:Female ratio | 1.59:1[c] | 1.73:1 |
| Total survival | 105 (92.1)[d] | 694 (77.4) |
| Histopathology | | |
| Nodular sclerosing | 76 (66.7)[e] | 467 (52.1) |
| Lymphocyte predominant | 15 (13.2) | 140 (15.6) |
| Mixed cellularity | 18 (15.8) | 228 (25.4) |
| Lymphocyte depleted | 2 (1.8) | 26 (2.9) |
| Unclassified | 3 (2.6) | 36 (4) |
| Staging | | |
| Stage I | 15 (13.2) | 173 (19.3) |
| Stage II | 47 (41.2) | 335 (37.3) |
| Stage III | 24 (21.1) | 160 (17.8) |
| Stage IV | 28 (24.6) | 229 (25.5) |

[a]Of 147 *DPB1* typed HD patients, 114 with relevant clinical data were compared with 897 from the same centre. [b]Data from 897 patients with Hodgkins disease patients presenting at the Department of Medical Oncology, Christie Hospital between 1974 and March 1995. [c]Male:female ratio for total patient series. [d]Figures in parentheses are percentage values.

### Data analysis

*DPB1* allele, phenotype and genotype frequencies in the patients and controls were computed, and results of phenotype frequency analysis compared by $\chi^2$ and Fisher's exact tests. Phenotype relative risks (RR) were calculated using the method of Sheehe (1966) and 95% confidence intervals (95% CI) of RRs calculated by the Mantel and Haentzel (1959) method using the CONTING, 2by2 and ReRi utilities for the IBM-PC provided by Professor Jurg Ott (Columbia University, NY, USA; see: http://linkage.rockefeller.edu/soft/linkutil). Significance values for the $\chi^2$ distribution and Fisher's exact test were subjected to the Bonferroni correction by multiplying *P*-values by the number of comparisons, unless otherwise indicated. Differences in *DPB1* phenotype distributions in HD subtype and control groups were determined using the log-likelihood ratio or *G*-test of Sokal and Rohlf (1981) as described by Klitz et al (1994). The *G*-statistic was calculated using a resampling computer algorithm (*rxc. exe*) written and provided by Dr George Carmody (Carleton University, Ottawa, Canada). The algorithm

 © 1999 Cancer Research Campaign

**Table 3** *HLA-DPB1* phenotype frequency in 147 patients with Hodgkin's disease analysed by histological subtype and gender

| DPB1 allele | All Hodgkin's disease (%) | | | | All control (%) | Male HD (%) | | | | Male control (%) | Female HD (%) | | | | Female control (%) |
|---|---|---|---|---|---|---|---|---|---|---|---|---|---|---|---|
| | Total | NS | LP | MC | | Total | NS | LP | MC | | Total | NS | LP | MC | |
| *0101 | 6.1 | 6.7 | 4.5 | 4.2 | 4.9 | 6.5 | 8.5 | 5.3 | 0 | 6.3 | 5.6 | 4.7 | 0 | 16.7 | 0 |
| *0201 | 11.6[a] | 12.2 | 4.5 | 12.5 | 21.3 | 10.8 | 12.8 | 5.3 | 11.1 | 16.1 | 13.0[b] | 11.6[c] | 0 | 16.7 | 33.3 |
| *0202 | 3.4 | 3.3 | 0 | 8.3 | 1.1 | 4.3 | 4.3 | 0 | 11.1 | 1.8 | 1.9 | 2.3 | 0 | 0 | 0 |
| *0301 | 27.2[d] | 28.9 | 27.3 | 33.3 | 20.8 | 22.6 | 25.5 | 21.1 | 27.8 | 23.2 | 35.2[e] | 32.6 | 66.7 | 50 | 17.6 |
| *0401 | 74.1 | 68.9 | 81.8 | 87.5 | 69.9 | 72.0 | 63.8 | 78.9 | 88.9 | 74.1 | 77.8 | 74.4 | 100 | 83.3 | 64.7 |
| *0402 | 15.6 | 16.7 | 22.7 | 8.3 | 22.4 | 17.2 | 17.0 | 26.3 | 11.1 | 24.1 | 13.0 | 16.3 | 0 | 0 | 23.5 |
| *0501 | 4.8 | 3.3 | 9.1 | 8.3 | 2.7 | 4.3 | 2.1 | 10.5 | 5.6 | 2.7 | 5.6 | 4.7 | 0 | 16.7 | 3.9 |
| *0601 | 3.4 | 4.4 | 0 | 4.2 | 3.3 | 2.2 | 2.1 | 0 | 5.6 | 1.8 | 5.6 | 7.0 | 0 | 0 | 7.8 |
| *0901 | 1.4 | 2.2 | 0 | 0 | 2.7 | 2.2 | 4.3 | 0 | 5.6 | 2.7 | 0 | 0 | 0 | 0 | 2.0 |
| *1001 | 3.4 | 5.6 | 0 | 0 | 2.2 | 1.1 | 2.1 | 0 | 0 | 1.8 | 7.4 | 9.3[f] | 0 | 0 | 0 |
| *1101 | 2.0 | 0[g] | 0 | 0 | 6.0 | 3.2 | 0 | 0 | 0 | 6.3 | 0 | 0 | 0 | 0 | 3.9 |
| *1301 | 4.1 | 5.6 | 0 | 4.2 | 2.2 | 4.3 | 6.4 | 0 | 5.6 | 2.7 | 3.7 | 4.7 | 0 | 0 | 0 |
| *1401 | 4.1 | 3.3 | 4.5 | 0 | 4.9 | 5.4 | 4.3 | 5.3 | 0 | 3.6 | 1.9 | 2.3 | 0 | 0 | 5.9 |
| *1501 | 2.0 | 1.1 | 4.5 | 4.2 | 0.5 | 2.2 | 0 | 5.3 | 5.6 | 0.9 | 1.9 | 2.3 | 0 | 0 | 0 |
| *1601 | 1.4 | 1.1 | 0 | 0 | 2.2 | 2.2 | 2.1 | 0 | 0 | 1.8 | 0 | 0 | 0 | 0 | 2.0 |
| *1701 | 1.4 | 2.2 | 0 | 0 | 2.2 | 2.2 | 4.3 | 0 | 0 | 1.8 | 0 | 0 | 0 | 0 | 2.0 |
| *1901 | 0.7 | 1.1 | 0 | 0 | 0.5 | 1.1 | 2.1 | 0 | 0 | 0.9 | 0 | 0 | 0 | 0 | 0 |
| *2001 | 1.4 | 0 | 9.1[h] | 0 | 0 | 2.2 | 0 | 10.5[i] | 0 | 0 | 0 | 0 | 0 | 0 | 0 |
| *2601 | 0 | 0 | 0 | 0 | 0.5 | 2.2 | 0 | 0 | 0 | 0.9 | 0 | 0 | 0 | 0 | 0 |
| *2901 | 0 | 0 | 0 | 0 | 0.5 | 2.2 | 0 | 0 | 0 | 0.9 | 0 | 0 | 0 | 0 | 0 |
| *3401 | 2.0 | 0 | 0 | 4.2 | 0.5 | 3.2 | 0 | 0 | 16.7[j] | 0.9 | 0 | 0 | 0 | 0 | 0 |
| *n* = | 147 | 90 | 22 | 24 | 183 | 93 | 47 | 19 | 18 | 112 | 54 | 43 | 3 | 6 | 51 |

| Footnote | DPB1 | Statistical comparison | Test | RR | 95% CI | Uncorrected *P*-value | Correction factor | Corrected *P*-value |
|---|---|---|---|---|---|---|---|---|
| a | *0201 | Total HD vs controls | $\chi^2$ | 0.49 | 0.27–0.90 | 0.0191 | 21 | >0.05 |
| b | *0201 | Female HD vs female controls | $\chi^2$ | 0.31 | 0.12–0.80 | 0.0130 | 42 | >0.05 |
| c | *0201 | Female NS-HD vs female controls | $\chi^2$ | 0.28 | 0.10–0.79 | 0.0133 | 126 | >0.05 |
| d | *0301 | Total HD vs controls | $\chi^2$ | 1.42 | 0.86–2.36 | 0.0171 | 21 | >0.05 |
| e | *0301 | Female HD vs female controls | $\chi^2$ | 2.46 | 1.02–5.92 | 0.0423 | 42 | >0.05 |
| f | *1001 | Female NS-HD vs female controls | Fisher's | 11.73 | 1.32–104.46 | 0.0405 | 252 | >0.05 |
| g | *1101 | NS-HD vs controls | Fisher's | 0.08 | 0.01–0.65 | 0.0111 | 126 | >0.05 |
| h | *2001 | LP-HD vs controls | Fisher's | 44.76 | 4.45–449.90 | 0.0111 | 126 | >0.05 |
| i | *2001 | Male LP-HD vs male controls | Fisher's | 32.14 | 3.17–326.17 | 0.0201 | 252 | >0.05 |
| j | *3401 | Male MC-HD vs male controls | Fisher's | 16.78 | 2.84–99.17 | 0.0083 | 252 | >0.05 |

Correction factors were applied to *P*-values as follows: x21: Number of phenotypes detected; x3: Number of histological subtypes; x2: Gender (male or female); x2: 2-tailed Fisher's exact test.

was set to simulate 10 000 random distributions in which two columns (patients and controls) were compared in a $2 \times N$ array, where $N$ = number of *DPB1* phenotypes (rows). *DPB1* allelic diversity ($h$) was calculated according to the method of Nei and Roychoudhury (1974) to provide an estimate of expected heterozygosity. This value was compared with the observed heterozygosity obtained from the frequency of each *DPB1* genotype.

### RESULTS

### Patients

Details of the patients, classified by histological subtype and gender, are shown in Table 1. Of the 147 patients, 90 (61.2%) were NS, 22 (14.9%) were LP, 24 (16.3%) were MC, one (0.68%) patient was LD and ten (6.8%) patients were of unclassifiable histological subtype. By gender, 50.5% of male patients compared with 79.6% of female patients were NS, 20.4% of males and 5.5% females were LP, and 19.3% of males and 11.1% of females were MC. Overall, male exceeded female patients by 37% (male:female ratio (M:F) = 1.72:1). The M:F for NS patients was 1.09, compared with 6.3 and 3.0 for LP and MC patients respectively. The one LD and ten unclassifiable patients were excluded from the DPB1 analysis of the non-NS patients, but were included in the overall analysis. Of the 22 LP patients, nine (40.9%) were classified as the nodular LP variant, but were not analysed separately from the other LP cases.

The mean and median ages of the total patient group were 33 years and 30 years respectively. The number of cases was highest in the 20- to 30-year age group, with an equal number of males and females in the 20- to 24-year group. Males exceeded females in most of the other age categories. The 19 male LP and 18 male MC cases were spread over the age range 15–49. Although a bimodal age distribution (Glaser and Jarrett, 1996) was not strongly evident, there was an increase in males > 60 years.

### Patient ascertainment

The patient group was a retrospective, hospital-based series referred for treatment and follow-up to a single centre. Patients were recruited to the HLA study either at presentation (i.e. before treatment) or during follow-up (i.e. at varying times after treatment) between 1990 and 1994. Patients presenting before this period, but lost to follow-up, were not included in the study. The

© 1999 Cancer Research Campaign

**Table 4** Frequency of DPB1 exon 2 polymorphic amino acids in 147 patients with Hodgkin's disease in relation to subtype and gender

| Amino acid | | All Hodgkins disease (%) | | | | All control (%) | Male HD (%) | | | | Male control (%) | Female HD (%) | | | | Female control (%) |
|---|---|---|---|---|---|---|---|---|---|---|---|---|---|---|---|---|
| Postn. | Residue | Total | NS | LP | MC | | Total | NS | LP | MC | | Total | NS | LP | MC | |
| 8 | Leucine | 89.8 | 86.7 | 100 | 95.8 | 92.3 | 89.2 | 83.0 | 100 | 100 | 92.9 | 90.7 | 90.7 | 100 | 83.3 | 96.1 |
| 8 | Valine | 49.7 | 52.2 | 50.0 | 45.8 | 45.9 | 47.3 | 48.9 | 47.4 | 44.4 | 49.1 | 53.7 | 55.8 | 66.7 | 50.0 | 37.3 |
| 9 | Phenylalanine | 89.8 | 86.7 | 100 | 95.8 | 92.3 | 89.2 | 83.0 | 100 | 100 | 92.9 | 90.7 | 90.7 | 100 | 83.3 | 96.1 |
| 9 | Histidine | 10.2 | 13.3 | 4.5 | 0 | 11.5 | 10.8 | 14.9 | 5.3 | 0 | 9.8 | 9.3 | 11.6 | 0 | 0 | 9.8 |
| 9 | Tyrosine | 44.2 | 44.4 | 45.5 | 45.8 | 36.1 | 40.9 | 38.3 | 42.1 | 44.4 | 40.2 | 50.0 | 51.2 | 66.7 | 50.0 | 27.5 |
| 11 | Glycine | 92.5 | 90.0 | 100 | 100 | 93.4 | 91.4 | 87.2 | 100 | 100.0 | 94.6 | 94.4 | 93.0 | 100 | 100 | 96.1 |
| 11 | Leucine | 44.2 | 47.8 | 40.9 | 41.7 | 41.5 | 40.9 | 44.7 | 36.8 | 38.9 | 43.8 | 50.0 | 51.2 | 66.7 | 50.0 | 37.3 |
| 35 | Phenylalanine | 98.6 | 98.9 | 95.5 | 100 | 99.5 | 98.9 | 100 | 94.7 | 100 | 99.1 | 98.1 | 97.7 | 100.0 | 100 | 100 |
| 35 | Leucine | 10.2 | 6.7 | 9.1 | 29.2[a] | 4.4 | 11.8 | 6.4 | 10.5 | 33.3[b] | 5.4 | 7.4 | 7.0 | 0 | 16.7 | 3.9 |
| 35 | Tyrosine | 14.3 | 13.3 | 9.1 | 12.5 | 13.7 | 16.1 | 14.9 | 10.5 | 11.1 | 16.1 | 11.1 | 11.6 | 0 | 16.7 | 3.9 |
| 36 | Alanine | 82.3 | 77.8 | 86.4 | 91.7 | 76.0 | 81.7 | 76.6 | 84.2 | 88.9 | 80.4 | 83.3 | 79.1 | 100 | 100 | 66.7 |
| 36 | Valine | 68.7 | 68.9 | 68.2 | 79.2 | 69.4 | 67.7 | 68.1 | 68.4 | 77.8 | 69.6 | 70.4 | 69.8 | 66.7 | 83.3 | 72.5 |
| 55 | Alanine | 83.7 | 77.8 | 86.4 | 100[c] | 76.5 | 83.9 | 76.6 | 84.2 | 100[d] | 81.3 | 83.3 | 79.1 | 100.0 | 100[f] | 66.7 |
| 55 | Aspartic acid | 61.9 | 64.4 | 63.6 | 58.3 | 67.2 | 61.3 | 66.0 | 63.2 | 55.6 | 67.0 | 63.0 | 62.8 | 66.7 | 66.7 | 70.6 |
| 55 | Glutamic acid | 8.8 | 7.8 | 9.1 | 16.7 | 4.4 | 9.7 | 8.5 | 10.5 | 16.7 | 5.4 | 7.4 | 7.0 | 0 | 16.7 | 3.9 |
| 56 | Alanine | 86.4 | 81.1 | 90.9 | 100[e] | 78.7 | 88.2 | 83.0 | 89.5 | 100 | 83.9 | 83.3 | 79.1 | 100 | 100 | 68.6 |
| 56 | Glutamic acid | 61.9 | 64.4 | 63.6 | 58.3 | 67.2 | 61.3 | 66.0 | 63.2 | 55.6 | 67.0 | 63.0 | 62.8 | 66.7 | 66.7 | 70.6 |
| 57 | Aspartic acid | 38.1 | 40.0 | 40.9 | 37.5 | 32.8 | 36.6 | 40.4 | 36.8 | 33.7 | 33.0 | 40.7 | 39.5 | 66.7 | 50.0 | 33.3 |
| 57 | Glutamic acid | 95.9 | 93.3 | 100 | 100 | 95.6 | 95.7 | 91.5 | 100 | 100 | 95.5 | 96.3 | 95.3 | 100 | 100 | 96.1 |
| 65 | Isoleucine | 93.9 | 94.4 | 100 | 91.7 | 95.6 | 92.5 | 93.6 | 100 | 88.9 | 96.4 | 96.3 | 95.3 | 100 | 100 | 96.1 |
| 65 | Leucine | 38.8 | 36.7 | 45.5 | 45.8 | 35.0 | 36.6 | 31.9 | 42.1 | 44.4 | 37.5 | 42.6 | 41.9 | 66.7 | 50.0 | 33.3 |
| 69 | Glutamic acid | 27.9 | 34.4 | 4.5[f] | 29.2 | 33.9 | 26.9 | 36.2 | 5.3[g] | 33.3 | 27.7 | 29.6 | 32.6 | 0 | 16.7 | 45.1 |
| 69 | Lysine | 95.2 | 93.3 | 100 | 100 | 93.4 | 95.7 | 93.6 | 100 | 100 | 95.5 | 94.4 | 93.0 | 100 | 100 | 92.2 |
| 69 | Arginine | 4.1 | 1.1 | 4.5 | 4.2 | 6.6 | 5.4 | 0 | 5.3 | 5.6 | 7.1 | 1.9 | 2.3 | 0 | 0 | 3.9 |
| 76 | Isoleucine | 4.8 | 6.7 | 0 | 4.2 | 2.7 | 5.4 | 8.5 | 0 | 5.6 | 3.6 | 3.7 | 4.7 | 0 | 0 | 0.0 |
| 76 | Methionine | 91.2 | 86.7 | 100 | 95.8 | 94.0 | 91.4 | 83.0 | 100.0 | 100 | 93.8 | 90.7 | 90.7 | 100 | 83.3 | 98.0 |
| 76 | Valine | 38.1 | 41.1 | 36.4 | 33.3 | 34.4 | 35.5 | 40.4 | 31.6 | 27.8 | 37.5 | 42.6 | 41.9 | 66.7 | 50.0 | 25.5 |
| 84 | Aspartic acid | 52.4 | 55.6 | 45.5 | 50.0 | 48.1 | 49.5 | 53.2 | 42.1 | 44.4 | 51.8 | 57.4 | 58.1 | 66.7 | 66.7 | 39.2 |
| 84 | Glycine | 86.4 | 85.6 | 90.9 | 87.5 | 89.1 | 83.9 | 80.9 | 89.5 | 44.4 | 90.2 | 90.7 | 90.7 | 100 | 83.3 | 92.2 |
| 84 | Valine | 4.1 | 1.1 | 4.5 | 16.7[h] | 1.1 | 5.4 | 0 | 5.3 | 22.2[i] | 1.8 | 1.9 | 2.3 | 0 | 0 | 0 |
| 85–87 | Glu-Ala-Val | 52.4 | 55.6 | 45.5 | 50.0 | 48.1 | 49.5 | 53.2 | 42.1 | 44.4 | 51.8 | 57.4 | 58.1 | 66.7 | 66.7 | 39.2 |
| 85–87 | Gly-Pro-Met | 87.8 | 85.6 | 90.9 | 95.8 | 89.6 | 86.0 | 80.9 | 89.5 | 100 | 91.1 | 90.7 | 90.7 | 100 | 83.3 | 92.2 |
| *n* = | | 147 | 90 | 22 | 24 | 183 | 93 | 47 | 19 | 18 | 112 | 54 | 43 | 3 | 6 | 51 |

| Footnote | Amino acid | | Statistical comparison | RR | 95% CI | Uncorrected *P*-value | Correction factor | Corrected *P*-value | Corrected Fishers *P* value |
|---|---|---|---|---|---|---|---|---|---|
| | Position | Residue | | | | | | | |
| a | 35 | Leucine | MC HD vs. controls | 8.85 | 3.04–25.77 | <0.0001 | 9 | 0.0001 | 0.0074 |
| b | 35 | Leucine | Male MC vs. male controls | 8.52 | 2.58–28.16 | 0.0001 | 18 | 0.0026 | >0.05 |
| c | 55 | Alanine | MC HD vs. controls | 15.17 | 2.00–115.20 | 0.0076 | 9 | >0.05 | 0.0459 |
| d | 55 | Alanine | Male MC vs. male controls | 8.69 | 1.10–68.49 | 0.0448 | 18 | >0.05 | >0.05 |
| e | 56 | Alanine | MC HD vs. controls | 13.40 | 1.76–101.91 | 0.0121 | 6 | >0.05 | >0.05 |
| f | 69 | Glutamic acid | LP HD vs. controls | 0.14 | 0.03–0.60 | 0.0048 | 9 | 0.0435 | 0.0402 |
| g | 69 | Glutamic acid | Male LP vs. male controls | 0.21 | 0.21–4.77 | 0.0355 | 18 | >0.05 | >0.05 |
| h | 84 | Valine | MC HD vs. controls | 15.94 | 3.55–71.49 | <0.0001 | 9 | 0.0002 | 0.0328 |
| i | 84 | Valine | Male MC vs. male controls | 13.72 | 2.97–63.32 | 0.0001 | 18 | 0.0023 | >0.05 |

Correction factors were applied to P values as follows: x2: Gender; x3: Histological subtypes; x2 or x3: Amino acid polymorphism; x2: 2-tailed Fisher's exact test.

minimum time interval from diagnosis to blood sampling was 1 week to 13.5 years (median 1.84 years). To assess the extent to which patient selection biased the *HLA* analysis, an *HLA*-typed patient group ($n$ = 114) for which there was detailed clinical data was compared with a total evaluable group of patients ($n$ = 897) attending the clinic between 1974 and 1995. The total evaluable group included the *DPB1*-typed patients.

The two groups are compared in Table 2, which shows that the distribution of HD subtypes was similar, although the frequency of NS was slightly greater and MC slightly less in the *HLA*-typed group compared with the total group. The M:F ratio of *DPB1*-typed patients was less than in the total group, reflecting a deficit of MC patients. The age and staging of the two groups were not remarkably different. The overall survival rate of the *HLA*-typed patients was 92.1% compared with 77.4% in the total evaluable group. Comparison of death rates in the total and *HLA*-typed groups by Cox regression analysis showed that the typed patients had a slightly lower death rate than the total group, but this was not significant ($P$ = 0.23). Given the favourable prognosis of HD in this centre (Radford et al, 1995), we conclude that any selection bias of the *HLA*-typed patients for survival was small.

### DPB1 allele and phenotype frequency analysis

*HLA-DPB1* typing revealed 19 alleles in the patient series and 20 alleles in the controls. One allele present in the patients was absent

© 1999 Cancer Research Campaign from the controls (**2001*), whilst two alleles (**2601*, **2901*) were absent from the patients but present in the controls. Analysis of allelic diversity (*h*) showed no significant difference in heterozygosity in the total HD group and the controls (data not shown).

The frequency of *DPB1* phenotypes in the total patient group and by subtype was compared with the controls using the $2 \times N$ log-likelihood ratio method to compute the *G*-statistic. No correction for the number of *DPB1* alleles is required with this method (Klitz et al, 1994). No significant difference was found in the total patient series or in each subtype alone (NS, LP and MC) compared with the controls (data not shown). However, the combined non-NS group (LP+MC+LD) showed a significant difference from the controls (*G*-statistic: 40.3, $P = 0.0053$), indicating a difference in the frequency distribution of *DPB1* phenotypes due to non-NS-HD.

To identify whether specific *DPB1* alleles were associated with susceptibility and resistance to HD, phenotype relative risks and 95% confidence intervals were determined allele-by-allele in $2 \times 2$ tests for each subtype and gender. The complete results, together with the statistical analysis, are shown in Table 3. They can be summarized thus: an excess of females of all HD subtypes typed for *DPB1*0301* (RR = 2.46; 95% CI 1.02–5.92); a deficit of all patients typed for *DPB1*0201* (RR = 0.49; 95% CI 0.27–0.90) and this was greater in females with NS-HD (RR = 0.28; 95% CI 0.10–0.79); in males, neither **0301* nor **0201* were associated with susceptibility or resistance respectively. In females, *DPB1*1001* was associated with resistance to NS (RR = 11.73; 95% CI 1.32–104.46), and **1101* with resistance to HD (RR = 0.08; 95% CI 0.01–0.65). In males, **2001* was associated with susceptibility to LP (RR = 32.14; CI 326.17), and **3401* was associated with susceptibility to MC (RR = 16.78; 95% CI 2.84–99.17).

### Analysis of polymorphic amino acids

*DPB1* alleles are composed of combinations of polymorphic DNA sequences in six HVRs in exon 2 which encode 2–3 polymorphic amino acids at each position. The identity of these amino acids can be predicted from the patterns of SSO hybridization used to assign classical *DPB1* alleles. Since none of the HVR sequences is allele-specific, the same sequence can occur in more than one classical *DPB1* allele. By comparing the frequency of polymorphic amino acids in patients and controls, corrections need only involve the number of amino acid alleles at that position. We therefore carried out a complete analysis in patients and controls of all polymorphic amino acids encoded at the following positions of *DPB1*: 8 (Leu/Val), 9 (Phe/His/Tyr), 11 (Gly/Leu), 35 (Phe/Leu/Tyr), 36 (Ala/Val), 55 (Ala/Asp/Glu), 56 (Ala/Glu), 57 (Asp/Glu), 65 (Ile/Leu), 69 (Glu/Lys/Arg), 76 (Ile/Met/Val), 84 (Asp/Gly/Val) and 85–87 (Glu-Ala-Val/Gly-Pro-Met).

The results are shown in Table 4 in relation to HD subtype and gender. After correction for the number of comparisons, four amino acid residues (leucine 35, alanine 55, alanine 56 and valine 84) were significantly associated with susceptibility to HD, and one (glutamic acid 69) was associated with resistance. Leu 35, Ala 55 and Val 84 were associated with susceptibility in males to MC (Leu 35: RR = 8.52, 95% CI 2.58–28.16; Ala 35: RR = 8.69, 95% CI 1.10–68.49; Val 84: RR = 13.72, 95% CI 2.97–63.32). Glutamic acid 69 was associated with resistance to LP (RR = 0.14; 95% CI 0.03–0.60) in males. In contrast to the classical *DPB1* phenotype analysis, none of the amino acid comparisons revealed significant associations with HD in females.

### DISCUSSION

A role for the HLA class II region and specifically for alleles at the *HLA-DPB1* locus in the aetiology of Hodgkin's disease has been proposed in a number of studies (Bodmer et al, 1989; Tonks et al, 1991, 1992; Klitz et al, 1994; Oza et al, 1994; Taylor et al, 1996). What has emerged from these studies is that *DPB1*0301* appears to confer susceptibility, and *DPB1*0201* resistance to HD. However, relative risks associated with these alleles were small, implying either that *DPB1* is only a minor contributor to HD susceptibility, or that they are masked by the diagnostic heterogeneity of the disease.

The present study extends our previous work (Taylor et al, 1996) by the addition of 29 patients and 91 adult controls, using a more detailed analytical approach. Our results, obtained with patients from a specific geographical region treated at a single specialist centre, confirm previous findings of an increase in *DPB1*0301* as an indicator of susceptibility (Tonks et al, 1992) and a decrease in **0201*, indicative of resistance (Bodmer et al, 1989) in HD. They also show that subclassification of HD by histological subtype *and* gender reveal additional associations which were not previously reported. Our results do not rule out a contribution from other *HLA class II* loci (Klitz et al, 1994) and, bearing in mind the greater incidence of non-NS-HD in males, a role for *HLA*-associated, X-linked susceptibility genes.

We found that the increase in *DPB1*0301* was greatest in females with non-NS, and the decrease in *DPB1*0201* was greatest in females with NS. Neither allele appeared to contribute to susceptibility or resistance to HD in males. Interestingly, susceptibility in males was associated with two rare alleles **2001* and **3401*, and involved LP and MC-HD respectively. We have previously reported a family with HD in two sisters, both of whom typed for *DPB1*2001* (Gokhale et al, 1995). In females, susceptibility to NS-HD was also associated with *DPB1*1001*. At first sight, these results appear to suggest that the HD subtype and the patient's gender might influence the *DPB1* association. However, the more likely explanation is that it is a person's gender and *DPB1* type influencing the HD subtype that develops following an aetiological event such as infection with a virus. If this is the case, it raises the possibility that certain *DPB1* alleles may do this by their influence on the presentation of infection-derived peptides to T-cells. Precisely how an ensuing immune response to the infectious agent affects HD pathology remains to be determined. One possibility is that the magnitude or type of T-cell response elicits the proliferation of a lineage-specific premalignant clone.

The present study was carried out on a hospital-based patient series which consisted of retrospectively and prospectively ascertained HD patients. We sought to minimize sources of bias by limiting the study to a single specialist treatment centre with diagnostic review by the same cancer pathology department. The excellent overall survival rate of the Manchester patients (Radford et al, 1995) suggests that there was only minimal selection bias favouring survivors. This was confirmed by comparing the survival of the *DPB1*-typed patients with a total evaluable patient group from the same centre. There was no major difference in overall survival despite a small deficit of MC patients in the *DPB1*-typed group. Previous studies of *HLA class II* alleles in HD patients (Tonks et al, 1992; Klitz et al, 1994) have also involved hospital-based patient series.

Although we tested and confirmed a prior hypothesis that *DPB1*0301* was associated with susceptibility and *DPB1*0201*

© 1999 Cancer Research Campaign 

with resistance to HD, the significance of these and other allele associations was lost when the null hypothesis of no association was corrected for the number of tests (*DPB1* alleles, HD subtype and gender). This emphasizes one of the problems encountered in studying the contribution of a highly polymorphic genetic system to the aetiology of a rare, diagnostically heterogeneous disease, especially where the genetic contribution may be indirect and of low penetrance. Even though uncorrected allele associations may be chance findings, they are of value in calculating the size of the patient and control groups required to verify such observations.

Since DPβ1 peptide diversity is determined by polymorphic amino acids coded by each of the six *DPB1* HVRs (Bugawan et al, 1990) an alternative approach was to analyse their frequency, instead of analysing classical *DPB1* alleles (i.e. **0101*, **0201* etc). Each HVR shows only limited polymorphism, so the magnitude of the Bonferroni correction at each position is much less than required when correcting for the number of classical *DPB1* alleles. Furthermore, since the polymorphic amino acids encoded by each HVR are themselves probably involved in determining antigenic peptide-binding and disease susceptibility, each polymorphic position in exon 2 is arguably at least, if not more, important than classical alleles.

The results of this analysis showed that even after correction for the number of comparisons (amino acid, subtype and gender), there were significant associations between specific amino acids and histological subtypes. Four amino acid residues were found to be involved in susceptibility to MC-HD, two of which (leucine 35 and valine 84) were significantly increased by virtue of their presence in rare *DPB1* alleles. The increased frequency of leucine 35 in males with MC can be related to its presence in *DPB1*0202*, **0501* and **3401*, whilst the increase in valine 84 in males with MC can be attributed to an excess of **1501* and **3401*. Alanine 55, which was increased in male MC, occurs in a number of alleles whose frequency was greater in males with MC (**0401*, **0301*, **1501* and **3401*) than in male controls. Glutamic acid at position 69 seems to protect males against LP, due to its occurrence in **0201*. Although not significant, there was also a reduced frequency of glutamic acid 69 in all female subtypes, and was completely absent (as was **0201*) from the three female LP patients. The allele **2001* occurred in two patients, both of which were males with LP, but not in controls. This rare allele is closely related to **0301*, with which it differs by coding methionine, not valine, at position 76. The occurrence of valine 76 in *DPB1*0201* suggests that HD susceptibility associated with **0301* and **2001* may not be due to this residue, since **0201* is associated with resistance to HD.

The elucidation of the three-dimensional structure of the DRβ1*0101 peptide (Brown et al, 1993) has identified regions of the β1 domain which are critical for anchoring processed antigenic peptides and for the stabilization of DRB α/β heterodimers. A comparison of DRβ1 with DPβ1 amino acid sequences shows 71% identity, whilst the remainder are highly homologous with respect to hydrophobicity. It is thus possible that the regions in DPβ1 equivalent to those in DRβ1 have a similar biological function. By alignment of DRβ1 and DPβ1 amino acid sequences, residue 55 of DPβ1 (equivalent to residue 57 of DRβ) may be in the P9 pocket, contributing to the stabilization of DPα/DPβ heterodimers by the formation of a salt bridge with DPα. Other homologies suggest that Val 84 corresponds to residue 86 of DRβ and contributes to the P1 pocket. Glutamic acid at position 69 corresponds to position 71 of DRβ and occurs in pocket 4. This pocket is involved in controlling antigen-specific T-cell responses (Fu et al, 1995) and is associated with susceptibility to tuberculoid leprosy (Zerva et al, 1996). A single amino acid change at position 71 of DRβ is sufficient to alter the peptide-binding characteristics and susceptibility to rheumatoid arthritis (Hammer et al, 1995), and clearly shows the close relationship between peptide binding and disease susceptibility. Furthermore, DPβglutamic acid 69 is strongly associated with susceptibility to hard metal lung disease (Potolicchio et al, 1997).

If HD is caused by a single type of infectious agent (EBV?), individual DPβ1 amino acid residues might influence interactions between premalignant cells containing the virus and host T-cells. The outcome of this interaction could then influence HD pathology during the transition to malignancy. Since EBV is expressed in Reed–Sternberg cells at higher frequency in MC than NS-HD, the increased frequency of leucine 35, alanine 55 and valine 84 in MC could indicate a direct role in interactions with viral peptides. The gender difference in the amino acid association could mean that the response to an aetiological agent is modified by X-linked genes. It is worth noting that the gene for X-linked lymphoproliferative disease, a disease associated with an inability to combat EBV infection, maps to Xq25 (Skare et al, 1989). A variant of the XLP gene, together with HLA-DPβ1 polymorphic amino acids, could perhaps contribute to susceptibility to MC-HD.

### ACKNOWLEDGEMENTS

We are grateful for the dedicated assistance of Diane Meynell and Amanda Watson during this project. The work was supported by grants from the Leukaemia Research Fund and Kay Kendall Leukaemia Fund to GM Taylor, and the Cancer Research Campaign to D Crowther.

### REFERENCES

Alexander FE, Ricketts TJ, McKinney PA and Cartwright RA (1991*a*) Community lifestyle characteristics and incidence of Hodgkin's disease in young people. *Int J Cancer* **48**: 10–14

Alexander FE, McKinney PA, Williams J, Ricketts TJ and Cartwright RA (1991*b*) Epidemiological evidence for the 'two-disease hypothesis' in Hodgkin's disease. *Int J Epidemiol* **20**: 354–361

Bodmer JG, Tonks S, Oza AM, Lister TA and Bodmer WF (1989) HLA-DP-based resistance to Hodgkin's disease. *Lancet* **i**: 1455–1456

Brown JH, Jardetzky TS, Gorga JC, Stern LJ, Urban RG, Strominger JL and Wiley DC (1993) Three-dimensional structure of the human class II histocompatibility antigen HLA-DR1. *Nature* **364**: 33–39

Bugawan TL, Begovich AB and Erlich HA (1990) Rapid HLA-DPB typing using enzymatically amplified DNA and nonradioactive sequence-specific oligonucleotide probes. *Immunogenetics* **32**: 231–241

Cesbron A, Moreau P, Rapp MJ, Cheneau ML, Herry P, Bonneville F, Muller JY, Harrouseau JL and Bignon JD (1993) HLA-DPB and susceptibility to Hodgkin's disease. *Hum Immunol* **36**: 51

Fu X-T, Bono CP, Woulfe SL, Swearingen C, Summers NL, Sinigaglia F, Sette A, Schwartz BD and Karr RW (1995) Pocket 4 of the HLA-DR (α,β*0401) molecule is a major determinant of T cell recognition of peptide. *J Exp Med* **181**: 915–926

Germain RN (1994) MHC-dependent antigen processing and peptide presentation: providing ligands for T lymphocyte activation. *Cell* **76**: 287–299

Glaser SL and Jarrett RF (1996) The epidemiology of Hodgkin's disease. *Ballière's Clin Haematol* **9**: 401–416

Gokhale DA, Evans DG, Crowther D, Woll P, Watson CJ, Dearden SP, Fergusson WD, Stevens RF and Taylor GM (1995) Molecular genetic analysis of a family with a history of Hodgkin's disease and dyschondrosteosis. *Leukemia* **9**: 826–833

Gutensohn N and Cole P (1977) Epidemiology of Hodgkin's disease in the young. *Int J Cancer* **19**: 595–604

 *© 1999 Cancer Research Campaign*

Gutensohn N and Cole P (1981) Childhood social environment and Hodgkin's disease. *N Engl J Med* **304**: 135–140
Hammer J (1995) New methods to predict MHC-binding sequences within protein antigens. *Curr Opin Immunol* **7**: 263–269
Hammer J, Gallazzi F, Bono E, Karr RW, Guenot J, Valsasnini P, Nagy ZA and Sinigaglia F (1995) Peptide binding specificity of HLA-DR4 molecules: correlation with rheumatoid arthritis association. *J Exp Med* **181**: 1847–1855
Jarrett RF (1993) Viruses and Hodgkin's disease. *Leukemia* **7**: S78–S82
Khan G, Coates PJ, Gupta RK, Kangro HO and Slavin G (1992) Presence of Epstein-Barr virus in Hodgkin's disease is not exclusive to Reed–Sternberg cells. *Am J Pathol* **140**: 757–762
Klitz W, Aldrich CL, Fildes N, Horning SJ and Begovich AB (1994) Localization of predisposition to Hodgkin's disease in the HLA class II region. *Am J Hum Genet* **54**: 497–505
Lukes RJ, Butler JJ and Hicks EB (1966*a*) Natural history of Hodgkin's disease as related to its pathologic picture. *Cancer* **19**: 317–344
Lukes RJ, Craver LF, Hall TC, Rappaport H and Ruben P (1966*b*) Report of the nomenclature committee. *Cancer Res* **26**: 1311
Marsh SG (1996) Nomenclature for factors of the HLA system, update May/June 1997: WHO Nomenclature Committee for Factors of the HLA system. *Tissue Antigens* **50**: 419–420
Mantel N and Haenszel W (1959) Statistical aspects of the analysis of data from retrospective studies of disease. *J Natl Cancer Inst* **22**: 719–748
Mauch PM, Kalish LA, Kadin M, Coleman CN, Osteen R and Hellman S (1993) Patterns of presentation of Hodgkin disease. *Cancer* **71**: 2062–2071
Miller G (1990) Epstein–Barr virus: biology, pathogenesis and medical aspects. In: *Field's Virology*, Fields BN, Snipe DM, Chanock RM et al (eds), pp. 1921–1958. Raven Press: New York.
Nei M and Roychoudhury AK (1974) Sampling variances of heterozygosity and genetic distance. *Genetics* **76**: 379–390
Oudejans JJ, Jiwa NM and Meijer CJLM (1997) Epstein-Barr virus in Hodgkin's disease: more than just an innocent bystander. *J Pathol* **181**: 353–356
Oza AM, Tonks S, Lim J, Fleetwood MA, Lister TA, Bodmer JG and Collaborating Centers (1994) A clinical and epidemiological study of human leukocyte antigen-DPB alleles in Hodgkins disease. *Cancer Res* **54**: 5101–5105
Pan LX, Diss TC, Peng HZ, Norton AJ and Isaacson PG (1996) Nodular lymphocyte predominance Hodgkin's disease: a monoclonal or polyclonal B-cell disorder? *Blood* **87**: 2428–2434
Pellegris G, Lombardo C, Cantoni A, Devizzi L and Balzarotti M. (1993) Study of the HLA-DPβ1 locus by the polymerase chain reaction technique in patients with Hodgkin's disease. *Tumori* **79**: 133–136
Potolicchio I, Mosconi G, Forni A, Nemery B, Seghizzi P and Sorrentino R (1997) Susceptibility to hard metal lung disease is strongly associated with the presence of glutamate 69 in HLA-DP beta chain. *Eur J Immunol* **27**: 2741–2743
Radford JA, Crowther D, Rohatiner AZS, Ryder WDJ, Gupta RK, Oza A, Deakin DP, Arnott S, Wilkinson PM, James RD, Johnston RJ and Lister TA (1995) Results of a randomized trial comparing MVPP chemotherapy with a hybrid regimen, Ch1VPP/EVA, in the initial treatment of Hodgkin's disease. *J Clin Oncol* **13**: 2379–2385
Sheehe PR (1966) Combination of log relative risk in retrospective studies of disease. *Am J Pub Health Nations Health* **56**: 1745–1750
Skare JC, Grierson HL, Sullivan JL, Nussbaum RL, Putilo DT, Sylla BS, Lenoir GM, Reilly DS, White BN and Milunsky A (1989) Linkage analysis of seven kindreds with the X-linked lymphoproliferative syndrome (XLP) confirms that the XLP locus is near DXS42 and DXS37. *Human Genet* **82**: 354–358
Sokal RR and Rohlf FJ (1995) *Biometry*, 3rd edn. WH Freeman: New York
Tait BD, Bodmer JG, Erlich HA, Ferrara GB, Albert E, Begovich A, Kimura A, Varney MD and Klitz W (1991) DNA typing: DPA and DPB analysis. In: *HLA 1991: Proceedings of the Eleventh International Histocompatibility Workshop and Conference*, Vol. 1. Tsuji K, Aizawa M and Sasazuki T (eds), pp. 485–496. Oxford Science: Oxford
Taylor GM, Gokhale DA, Crowther D, Woll P, Harris M, Alexander F, Jarrett R and Cartwright RA (1996) Increased frequency of HLA-DPB1*0301 in Hodgkin's disease suggests that susceptibility is HVR-sequence and subtype associated. *Leukemia* **10**: 854–859
Tonks S, Oza AM, Lister TA, Bodmer JG and Collaborating Centres (1991) An international study of the association between HLA-DP and Hodgkin's disease. In: *HLA 1991: Proceedings of the Eleventh International Histocompatibility Workshop and Conference*, Vol. 2. Tsuji K, Aizawa M and Sasazuki M (eds), pp. 539–544. Oxford Science: Oxford
Tonks S, Oza AM, Lister TA and Bodmer JG (1992) Association of HLA-DPB with Hodgkin's disease. *Lancet* **i**, 340: 968–969
Vianna NJ, Greenwald P and Davies JNP (1971) Nature of Hodgkin's disease agent. *Lancet* **i**: 733–736
Vianna NJ (1974) Is Hodgkin's disease infectious? *Cancer Res* **34**: 1149–1155
Weiss LM, Movahed LA, Warnke RA and Sklar J (1989) Detection of Epstein–Barr viral genomes in Reed-Sternberg cells of Hodgkin's disease. *N Engl J Med* **320**: 502–506
Wu T-C, Mann RB, Charache P, Hayward SD, Staal S, Lambe BC and Ambinder RF (1990) Detection of EBV gene expression in Reed-Sternberg cells of Hodgkin's disease. *Int J Cancer* **46**: 801–804
Zerva L, Cizman B, Mehra NK, Alahari SK, Murali R, Zmijewski CM, Kamoun M and Monos DS (1996) Arginine at positions 13 or 70–71 in pocket 4 of HLA-DRB1 alleles is associated with susceptibilty to tuberculoid leprosy. *J Exp Med* **183**: 829–836

*© 1999 Cancer Research Campaign*
